# Traumatic inflammatory response: pathophysiological role and clinical value of cytokines

**DOI:** 10.1007/s00068-023-02388-5

**Published:** 2023-12-27

**Authors:** Rui Li, Jing Jing Ye, Lebin Gan, Mengwei Zhang, Diya Sun, Yongzheng Li, Tianbing Wang, Panpan Chang

**Affiliations:** 1 Trauma Medicine Center, Peking University People’s Hospital, Beijing, 100044 People’s Republic of China; 2 Key Laboratory of Trauma and Neural Regeneration (Peking University) Ministry of Education, Beijing, 100044 People’s Republic of China; 3 National Center for Trauma Medicine of China, Beijing, 100044 People’s Republic of China; 4 Biomedical Pioneering Innovation Center (BIOPIC), Peking University, Beijing, People’s Republic of China

**Keywords:** Trauma, Inflammatory response, Cytokine, Pathophysiological mechanisms, Diagnosis and treatment

## Abstract

Severe trauma is an intractable problem in healthcare. Patients have a widespread immune system response that is complex and vital to survival. Excessive inflammatory response is the main cause of poor prognosis and poor therapeutic effect of medications in trauma patients. Cytokines are signaling proteins that play critical roles in the body's response to injuries, which could amplify or suppress immune responses. Studies have demonstrated that cytokines are closely related to the severity of injuries and prognosis of trauma patients and help present cytokine-based diagnosis and treatment plans for trauma patients. In this review, we introduce the pathophysiological mechanisms of a traumatic inflammatory response and the role of cytokines in trauma patients. Furthermore, we discuss the potential of cytokine-based diagnosis and therapy for post-traumatic inflammatory response, although further clarification to elucidate the underlying mechanisms of cytokines following trauma is warranted.

## Background

Severe trauma remains the leading cause of mortality and disability in young adults [1]. According to the World Health Organization, injuries account for 8% of global deaths, and in 2019, more than 4 million people died due to injuries [2]. Furthermore, the treatment for patients with severe trauma is intensive and long-lasting, contributing to significantly increased healthcare costs [3]. Although the healthcare field primarily focuses on preventing and treating cancer and cardiovascular and cerebrovascular diseases, traumatic injuries have become a substantial threat that endangers people’s property and life.

In the wake of the Second World War, the rapid evolution of medical technology and the refinement of therapeutic strategies for hemorrhagic and coagulation disorders have significantly augmented the prospects for early-stage efficacious patient interventions. Nonetheless, notwithstanding the enhanced survivability achieved in the initial phases of medical care, patients afflicted by severe trauma continue to confront substantial mortality risks post-hospital admission. Presently, the principal determinants contributing to the adverse prognosis of severe trauma patients subsequent to admission encompass the development of multiple organ dysfunction syndrome (MODS) and nosocomial infections. Crucially, the post-traumatic inflammatory response emerges as the pivotal factor underpinning the occurrence of MODS and nosocomial infections, garnering escalating attention from the scientific community [4–6].

Cytokines play an important role in the inflammatory response of the body [7, 8]. The balance of pro-inflammatory and anti-inflammatory cytokines is critical for the immune system. Recent years, accumulating studies explore the underlying mechanisms of cytokines following trauma. Studies have shown that cytokines are closely related to the severity of injuries and prognosis of trauma patients [9–11]. Some studies present the potential of cytokine-based diagnosis and treatment for trauma patients [12, 13]. Cytokines have been receiving increased attention in healthcare research. However, both of the mechanisms and clinical application of cytokines in trauma are still needed to be further studied.

The review initiated with a systematic search of the PubMed database using the keywords "trauma" and "cytokines". Subsequently, we conducted a comprehensive examination of full-text articles spanning the past 2 decades to synthesize the content of this article. Within the context of this systematic review, we meticulously elucidated the etiology of post-traumatic inflammation, with a specific emphasis on the central role played by cytokines in orchestrating this intricate physiological response. Furthermore, we provided a summary of the potential applications of cytokines in diagnosing and treating post-traumatic inflammation. Lastly, we outlined the current state of cutting-edge research in this field and provided insights into future research directions.

## Review

### Pathophysiological mechanisms of traumatic inflammatory response

In a post-traumatic context, there emerges a dichotomous immune response characterized by an initial nonspecific activation followed by a prolonged immunosuppressive phase. This biphasic immune modulation predisposes trauma patients to not only autologous tissue insult and multi-organ dysfunction but also an amplified risk of infectious complications, including sepsis.

#### Systemic inflammatory response syndrome (SIRS)

After acute severe trauma, there is a pronounced alteration in the body's internal physiological milieu over a remarkably brief duration. In the wake of cellular perturbation and pronounced hemorrhagic events, there is a consequential augmentation in the release of damage-associated molecular patterns (DAMPs). Concomitantly, the human physiologic response orchestrates an intricate inflammatory cascade, strategically serving both to bolster defense mechanisms and expedite tissue reparative processes. Coinciding with this, the magnitude of the trauma triggers an expedited reaction from both the coagulative and neuroendocrine mechanisms. This phenomenon results in a persistent amplification of the inflammatory process due to a prevailing positive feedback loop. An elevated release of pro-inflammatory mediators compromises the integrity of endothelial cells, facilitating an influx of these mediators into the systemic circulation. The presence of circulating pro-inflammatory cytokines exacerbates endothelial damage, catalyzing an augmented release of inflammatory agents, thereby precipitating a pronounced instance of SIRS. Within the context of the SIRS, there is a discernible elevation in circulating pro-inflammatory cytokines coupled with the pronounced activation of immune cells. SIRS can be delineated as a manifestation of immunological imbalances, there exists a pronounced immunological disequilibrium characterized by the perturbation of pro-inflammatory and anti-inflammatory pathway equilibrium. This imbalance fosters a heightened sensitivity within the immune architecture, thereby amplifying the host's vulnerability to infectious agents and predisposing them to septic complications. Exaggerated inflammatory reactions, coupled with anomalies in coagulation, fibrinolytic dysfunctions, and the compounded interplay among various organ systems culminate in multi-organ failure. Such physiological disarray directly correlates with heightened mortality rates in affected individuals. To encapsulate, the volatile dynamics of the inflammatory response in the immediate post-traumatic phase are critical factors that impact patient outcomes [5, 14].

#### SIRS and compensatory anti-inflammatory response syndrome (CARS)

In tandem with the onset of the SIRS, the body concurrently activates the CARS as a counterbalance [15]. CARS is a deactivation of the immune system tasked with restoring homeostasis which involves the release of anti-inflammatory mediators and suppression of pro-inflammatory cytokines [16]. A delicate equilibrium exists between the pro-inflammatory (SIRS) and anti-inflammatory (CARS) responses. An optimal balance ensures tissue repair, clearance of pathogens, and prevention of excessive inflammation. However, if CARS predominates or is prolonged, it can lead to an immune-suppressive state. A potent or extended CARS response can render the host more susceptible to secondary infections, as the immune system's defense mechanisms are dampened. There can also be delayed wound healing, difficulty in clearing primary infections, and a heightened risk for hospital-acquired or opportunistic infections [16, 17].

DAMPs derived from cytosolic, nuclear, and mitochondrial sources are widely recognized as pro-inflammatory agents, however, emerging research suggests their significant involvement in immunosuppressive mechanisms. These DAMPs possess the ability to curtail the antimicrobial actions spanning both innate and adaptive immune spectra [18, 19]. For example, research findings have revealed that certain DAMPs, such as S100 proteins, possess immunosuppressive properties. Austermann et al. posited that S100A8/9 possesses the capability to attenuate monocyte functionality, thereby exerting immunosuppressive effects [20]. “S100A8/9 not only exerts a direct inhibitory influence on the functionality of infiltrating leukocytes but also recruits myeloid-derived suppressor cells (MDSCs), thereby further enhancing the immunosuppressive milieu [21]. MtDNA is also a recognized pro-inflammatory mediator, however, data are emerging that demonstrates mtDNA is a potent immune suppressor [22, 23]. Further complicating the immunosuppressive scenario, both at localized and systemic levels, is the consequence of the immediate release of DAMPs from injured tissues. These ancillary processes, including fluid resuscitation, surgical interventions, and the transfusion of aged blood derivatives, contribute to this complexity [24].

### Role of cytokines in the inflammatory response of patients with severe trauma

#### Cytokines

Cytokines are signaling proteins that play key roles in the body's response to injuries and amplify or suppress the immune responses directly [25].

Cytokines are small proteins with extensive biological activity. They are synthesized and secreted by immune cells, including monocytes, macrophages, T-cells, B-cells, natural killer (NK) cells, and some non-immune cells, including endothelial cells, epidermal cells, fibroblasts, and glial cells [26, 27]. Cytokines can be classified in various methods [28]. According to their structure and function, cytokines are divided into various subtypes, including IL, colony-stimulating factor (CSF), interferon (IFN), tumor necrosis factor (TNF), growth factor (GF), and chemokines [29]. IL regulates leukocytes. CSF can stimulate the differentiation and proliferation of pluripotent hematopoietic stem cells and progenitor cells. The main functions of IFN include anti-viral, anti-tumor, and anti-cell proliferation activities and immune regulation. TNF can regulate the immune response, kill target cells, and induce apoptosis. GF can promote cell growth and differentiation. Finally, chemokines promote chemotaxis and recruitment of various cell types, including leukocytes [29]. They bind to specific receptors on the surface of target cells and initiate a series of intracellular signal transduction pathways through three modes of action: autocrine (acting on the secretory cell itself), paracrine (acting on the peripheral of the secretory cell), and endocrine (acting at a distance through blood circulation) [30]. Cytokines act similar to hormones, except that cytokine release can lead to cascades of subsequent cytokines and receptors in other cells or can establish feedback loops [31]. Cytokines have short half-lives, which normally prevents them from having effects outside lymphoid tissue and sites of inflammation [32]. Their early role in the inflammatory response can be classified as pro- or anti-inflammatory, promoting or inhibiting the occurrence and development of an inflammatory response [33]. Normally, the body regulates these cytokines such that both the release of cytokines and recruitment of immune cells are controlled at the local injury site, which is beneficial for body defense and tissue repair.

**Fig. 1:**
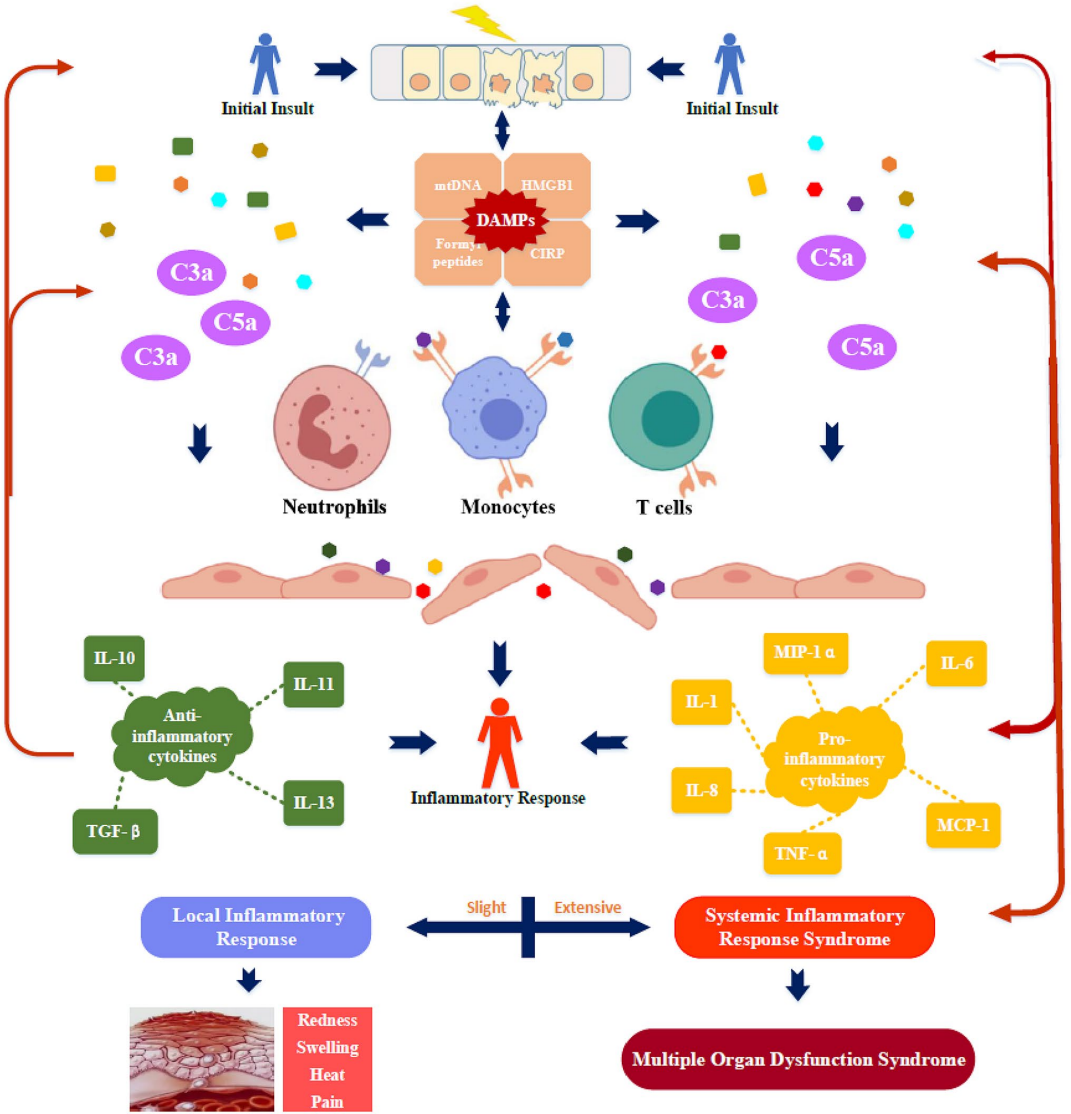
Initial insult causes a massive release of DAMPs that trigger an inflammatory response. DAMPs stimulate the cells of the innate immune system and activate the complement cascade [5, 148]. The activation of complement and inflammatory cells triggers the production and release of cytokines [16]. Cytokines play critical roles in the immune response [39]. Slight inflammatory reactions are controlled locally and manifest as redness, swelling, heat, and pain at the site of injury. Extensive inflammatory reactions may cause SIRS, which can lead to MODs [149]. Tissue damage leads to the release of additional DAMPs and cytokines, resulting in a vicious cycle with continued immune activation [5]. *mtDNA* Mitochondrial DNA, *HMGB1* High mobility group box protein 1, *DAMPs* Damage-associated molecular patterns, *CIRP* Cold-inducible RNA-binding protein, *IL* Interleukin, *MCP-1* Monocyte chemoattractant protein 1, *MIP* Macrophage inflammatory protein, *TGF* Transforming growth factor, *TNF* Tumor necrosis factor, *C* complement

However, even though they play a protective role in patients with severe trauma, they also aggravate damage to the body [7, 34]. Studies have shown that cytokines are closely related to the occurrence and development of severe inflammatory reactions and complications in patients with severe trauma [35, 36]. In patients with severe trauma and hemorrhagic shock, a sudden excessive release of cytokines occurs throughout the body, and loss of endothelial integrity enables cytokines to affect tissues far from the site of the original injury [37, 38]. The inordinate release of these cytokines, as well as their unpredictable interactions, trigger and aggravate SIRS, which may lead to MODS or even death [39, 40] (Fig. 1). This is a phenomenon of excessive immunity produced in response to external stimuli [25]. Patients at this stage may have fatigue, anorexia, headache, rash, diarrhea, arthralgia, myalgia, and neuropsychiatric findings. Cases can progress rapidly to disseminated intravascular coagulation with either vascular occlusion or catastrophic hemorrhages, dyspnea, hypoxemia, hypotension, hemostatic imbalance, vasodilatory shock, and death [25]. Some other diseases have similar clinical features; for example, some studies on COVID-19 have stated that excessive inflammatory response is the main cause of death [25, 41]. Some researchers refer to this condition as a “cytokine storm” or "cytokine release syndrome (CRS)” terms that are not yet accepted universally in the field of trauma. Unfortunately, owing to a lack of understanding of the corresponding pathophysiological mechanisms, targeted treatment in this field is limited.

#### Pro-inflammatory cytokines

Pro-inflammatory cytokines control inflammatory responses. At the injury site, pro-inflammatory cytokines inform the surrounding cells and tissues and induce acute responses to protect the host and repair injury by activating neutrophils, endothelial cells, and epithelial cells. Simultaneously, cytokines can enter circulation and activate immune cells [42]. However, cytokines may bring large-scale damage to multiple tissues and organs, and some cytokine-related instances have been reported to be associated with death after severe injury. As for the regulation and function of IL-6 (PMID: 25,190,079; PMID: 25,898,198), macrophage inflammatory protein (MIP)-1α, TNF-α, IL-1, IL-8, and monocyte chemoattractant protein-1 (MCP-1) have been perfectly discussed in previous works, and our review mainly focuses on the roles of these cytokines in trauma [43] (Table 1).

**Table 1:** Cytokines with pro-inflammatory activities

Cytokines	Source	Major activities
IL-6 [15, 24, 25]	Monocytes, macrophages, endothelial cells, epithelial cells, and fibroblasts	Pro-inflammatory cytokines, increase antibody production, induce acute-phase reactants pyrogenic function, promote the differentiation of B-cells into plasma cells, enhance T-cell proliferation, and have an anti-inflammatory function
MIP-1α [32, 33]	Monocytes, neutrophils, dendritic cells, NK cells, and mast cells	Recruit macrophages for Th1, NK, eosinophil, and dendritic cells and have a pyrogenic function
TNF-α [39–41]	Monocytes, macrophages, T-cells, B-cells, NK cells, mast cells, and dendritic cells	Increase vascular permeability and pyrogenic function
IL-1 [47]	Monocytes, macrophages, dendritic cells, NK cells, and epithelial cells	Pro-inflammatory alarmin cytokine, promote T-cell activation, induce acute-phase reactants, promote B-cell proliferation and maturation, have a pyrogenic function, and activate macrophage and Th17 cell activation
IL-8 [55, 56]	Monocytes, macrophages, epithelial cells, and endothelial cells	Recruit neutrophils
MCP-1 [58, 59]	Macrophages, dendritic cells, and cardiac myocytes	Recruit Th2 cells, monocytes, dendritic cells, and basophils
IL-22 [17, 80–83]	T-cells	Production of antibacterial proteins, protective role against pathogens, and tissue regeneration

*IL* interleukin, *MCP* monocyte chemoattractant protein, *MIP* macrophage inflammatory protein, *TGF* transforming growth factor, *TNF* tumor necrosis factor

#### IL-6

IL-6 is a 21-kDa glycoprotein with primarily pro- and some anti-inflammatory effects [27]. During the acute phase of the post-traumatic inflammatory response, specific macrophages, including Kupffer cells, secret IL-6 and stimulate the synthesis and release of acute-phase proteins by liver cells, consequently enhancing the systemic inflammatory response. Subsequently, IL-6 can play an anti-inflammatory role by downregulating pro-inflammatory factors, including IL-1 and TNF-α [44, 45]. Studies have shown that the level of IL-6 in the plasma of injured patients is closely related to the severity of trauma and positively correlates with the incidence and mortality of MODS [46–48]. Specifically, in a prospective cohort study, Stensballes et al. reported that the serum level of IL-6 during the early systemic inflammatory response exhibited strong positive correlations with trauma severity and 30-day mortality. A strong correlation between the Injury Severity Score (ISS) and IL-6 was observed [47]. Kumari et al. reported that IL-6 and IL-1β were significantly more expressed in patients with blunt chest trauma who had a fatal outcome during hospital stay than those who had a good prognosis [9]. When comparing the serum levels of cytokines between patients who died of trauma and those who died of others causes, the levels of IL-6 and IL-8 were significantly higher in the former population than in the latter population, associating IL-6 and IL-8 with injury. Moreover, IL-6 and IL-8 levels are important biomarkers for distinguishing traumatic from non-traumatic death, assessing trauma severity, and supporting the diagnosis of traumatic death [49]. Joseph et al. analyzed the plasma IL-6 levels of patients with severe trauma accompanied by hemorrhagic shock and concluded that the level of plasma IL-6 was related to the MODS score and incidence, duration of ventilator use, intensive care unit (ICU) hospitalization, and prognosis. Furthermore, Clausen et al. found that post-traumatic brain tissue and plasma levels of IL-6 were elevated in a rat model of TBI [50]. Additionally, in a study by Homeier et al., sgp130Fc, a selective inhibitor of the IL-6 pathway, was used as an intervention strategy in mice with multiple injuries. Results have suggested that sgp130Fc could inhibit the release of IL-6; however, the inhibition of the IL-6 pathway alone had no effect on the release of other inflammatory factors in severely injured mice and no significant effect on mortality [11]. The liver is the main source and scavenging organ of IL-6. By investigating patients with liver ischemia–reperfusion injury, Clavien et al. reported that, as IL-6 becomes more expressed, the synthesis of TNF-α gradually decreases, and the self-synthesized IL-6 could effectively control the inflammatory response to liver ischemia–reperfusion injury. Thus, the protective effect of IL-6 on the liver is achieved by the downregulation of TNF-α [51]. The above-mentioned data indicate that the inhibition of IL-6 alone has a limited effect on the systemic immune response. The synergistic effect of IL-6 and other cytokines is more likely to affect SIRS and warrants further research.

#### MIP-1α

MIP-1α is an important chemotactic cytokine involved in the immune responses. It is released in large quantities during hypoxia, ischemia, infection, and immune responses after severe trauma and plays a role in the systemic inflammatory response after traumatic bleeding [52, 53]. MIP-1α can activate and induce circulation of macrophages, NK cells, dendritic cells, eosinophils, lymphocytes, and mast cells to induce IL-1, IL-6, TNF-α, and other inflammatory mediators. Additionally, MIP-1α can induce NK cells to participate in the inflammatory response and activate B-cells to produce IgE and IgG antibodies. Moreover, MIP-1α is secreted by various cell types including macrophages, lymphocytes, and endothelial cells. Furthermore, Hsieh et al. found that MIP-1α plays an important role in the acute inflammatory response induced by post-traumatic hemorrhage, and when MIP-1α is inhibited, the acute inflammatory response is attenuated. Therefore, reducing MIP-1α levels in patients with traumatic hemorrhagic shock may reduce the inflammatory response to trauma [54]. MIP-1α also plays an important role in brain injury and indicates its severity [53]. The secretion and expression of MIP-1α in brain tissue is low under normal physiological conditions, but a significant amount of MIP-1α is secreted and released in the cerebrospinal fluid under stress, with a small amount entering the bloodstream [55]. During TBI, the microglia, astrocytes, and neurons in the brain are affected by ischemia, hypoxia, and severe damage. Using a mouse model, Ciechanowska et al. found that the expression of MIP-1α family members (CCL3, CCL4, and CCL9), as well as MIP-1α receptors (CCR1 and CCR5), increased in the brain tissue of mice with TBI. Increased MIP-1α promotes neutrophil recruitment and the activation of microglia and astrocytes, suggesting that the MIP-1α family is an important target for drug intervention after brain injury [56]. MIP-1α can also regulate the growth of hematopoietic stem cells and hematopoietic progenitor cells. The related research on leukemia has demonstrated that MIP-1α can inhibit the proliferation of hematopoietic stem cells [57]. Whether this function plays a role in severe trauma and massive blood loss requires further investigation. Finally, MIP-1α plays an important role in hemorrhagic shock and craniocerebral injury, two major lethal factors after severe trauma. Briefly, clarifying the mechanism of MIP-1α in trauma is an ongoing focus of research on trauma intervention.

#### TNF-α

TNF-α is one of the earliest-to-be-expressed and most powerful cytokines in the inflammatory response and is mainly produced by monocytes and macrophages. As an upstream factor, TNF-α can activate the NF-kβ, MAPK, and Fas-FasL signaling pathways, among others, to regulate downstream inflammatory factors, including IL-1, IL-6, and IL-8 [58, 59]. TNF-α can induce the activation and aggregation of neutrophils in the early stage of inflammation, enhance the activity and killing function of monocytes and macrophages, and promote the removal of damaged and necrotic tissue cells [60, 61]. In a mouse model of trauma, the treated mice demonstrated significantly higher levels of TNF-α in the blood compared with the control mice [62]. Palmer et al. analyzed blood samples from trauma patients and found that TNF-α levels were higher and more pronounced [63]. Additionally, traumatic hemorrhagic shock can lead to visceral injury, especially liver ischemia–reperfusion injury. Researchers have found that in the initial stage of liver ischemia–reperfusion injury, excessive TNF-α is produced, resulting in organ damage. The inhibition of TNF-α activation down-regulates NF-κB, thereby reducing the accumulation of neutrophils and inhibiting the production of inflammatory cells. Local infiltration of TNF-α can play a role in reducing the damage of the inflammatory response [64]. Overall, TNF-α has a wide range of effects and can impact the functions of the circulatory system, cardiovascular system, liver, kidney, and other organs. TNF-α is also closely related to sepsis, heart failure, and organ transplant rejection [65, 66]. However, due to its broad role and lack of specificity, TNF-α is usually not used as a biomarker for disease diagnosis and identification. Nevertheless, it can be used to evaluate the treatment effect and prognosis of certain diseases, suggesting that it may be useful as an index to evaluate SIRS severity in trauma patients.

#### IL-1

The IL-1 family includes IL-1α and IL-1β, which are produced by macrophages and neutrophils. IL-1α and IL-1β act on the same receptors related to innate immunity. The function of IL-1 is thought to be similar to that of DAMPs. It acts as a pro-inflammatory cytokine produced in the early stage of trauma and cascades the inflammatory process. IL-1 also induces immune cells to produce cytokines and other inflammatory mediators, leading to uncontrolled inflammatory responses and cytokine storms, ultimately causing tissue and organ damage [67]. Clausen et al. used a rat model to study TBI and found that IL-1 levels were elevated in both brain and plasma after trauma [50]. Additionally, a study on patients with burns found that the serum levels of IL-1 significantly increased 6–24 h following the burn injury. By monitoring the changes in IL-1α throughout the course of SIRS, patients with hypovolemic shock in the late stage were found to have higher levels of IL-1α than did those with a favorable prognosis [68]. Severe trauma is often accompanied by massive blood loss and ischemia–reperfusion injury to organs after blood loss, which is one of the main causes of mortality. In a study on myocardial reperfusion injury, Mauro et al. found that ischemia–reperfusion injury can lead to inflammasome formation, whereas blocking IL-1α can significantly reduce inflammasome formation and inhibit inflammasome activation. Consequently, blocking IL-1α protects the heart by reducing infarct size and preserving left ventricular function [69]. Therefore, IL-1α blockade may be a new therapeutic strategy to reduce ischemia–reperfusion injury. Nevertheless, whether IL-1 blockade can also protect other important organs in trauma patients, including the liver and kidney, requires further exploration. Investigations of IL-1 could provide implications for the clinical decision-making of organ ischemia–reperfusion injury. Moreover, Anja et al. found that IL-1 plays an important role in initiating emergency hematopoiesis after trauma [70]. Microglia are thought to be sensors of brain injury during SIRS. Moreover, after acute TBI, microglia secrete excessive amounts of IL-1 [71, 72], which have been found to stimulate astrocyte growth and promote neovascularization at the wound site [73, 74]. Additionally, after injury, microglia is more expressed. In conclusion, IL-1 plays an important role in various complications of severe trauma.

#### IL-8

IL-8 is generally considered to be a pro-inflammatory cytokine that exerts chemotactic effects, attracts and activates neutrophils to the site of injury, and promotes neutrophil degranulation by acting through the release of various enzymes, including proteases [75]. Fumiko et al. studied skin wounds and found that injury can induce increased expression of IL-8, increasing the local inflammatory response. They also observed that aseptic inflammation caused by mechanical factors (friction/extrusion) can promote IL-8 production [76]. Additionally, increased levels of IL-8 in cerebral endothelial cells have been observed in studies on brain injury and inflammation and have been shown to affect blood–brain barrier permeability, infarct size, and edema after TBI [77]. Together, these findings indicate that the role of IL-8 after trauma is related to chemotaxis and early inflammatory response. Moreover, excessive secretion of IL-8 may lead to a significant local inflammatory response, resulting in more damage than protection.

#### MCP-1

MCP-1 can promote the chemotaxis of inflammatory cells during inflammation, as well as the expression of various inflammatory factors. MCP-1 activates monocytes in the early stage of the inflammatory response, activates and expands the inflammatory response, and has chemotactic effects on monocytes, basophils, activated NK cells, and memory T lymphocytes. Moreover, MCP-1 induces mast cell and basophil degranulation to release active substances, including histamine, thereby affecting the degree of the local inflammatory response [78, 79]. Dalgard et al. observed increased production of circulating MCP-1 in trauma patients and assessed this as a candidate biomarker for chronic post-traumatic stress disorder [79]. Additionally, Xu et al. found that after renal ischemia–reperfusion injury, inflammatory cells promote MCP-1 production through granulocyte–macrophage colony-stimulating factor, producing a sustained inflammatory response that damages renal tubules and, consequently, leading to local fibrosis [80]. MCP-1 can also induce and regulate the expression of cytokines, including IL-1 and IL-6. It also plays a key role in various sterile inflammatory processes resulting from cardiovascular disease, brain disease, bone and joint disease, tumors, and endothelial dysfunction [79]. Finally, in the aforementioned study on brain injury and inflammation, researchers found that MCP-1 plays an important role in the permeability of the blood–brain barrier, infarct size, and occurrence of edema after TBI [77]. Therefore, MCP-1 is not only a chemotactic substance but also a cytokine that regulates cell function. Currently, relatively few studies have evaluated MCP-1.

#### IL-22

IL-22 is a multifunctional cytokine acting on trauma inflammation, wound healing, and tissue regeneration and against pathogens [81]. IL-22 is mainly produced by T lymphocytes, specifically CD4 + T-cells or T helper cells. It is produced immediately to act against tissue impairments through the innate immune response [82]. IL-22 could stimulate the production of chemokines, such as C-X-C ligand (CXCL) 1 and CXCL2, and recruit neutrophils as an inflammatory response for tissue damage [83]. The IL-9 and IL-22 cytokines were found to be significantly more expressed in patients with blunt chest trauma than in healthy controls, but there was no significant correlation with patient outcomes [9]. IL-22 is of interest to researchers owing to its tissue-related protective and reparative functions. IL-22 could activate various downstream signaling pathway closely associated with trauma response, including the innate immune response (IL-6, TNF-a, CXCL and IL-8), protections against infections (S100 calcium binding protein A (S100A)7, S100A8, S100A9, and beta defensing-2), and tissue regeneration (B-cell lymphoma-2(bcl-2) family, vascular endothelial growth factor (VEGF), and matrix metalloproteins (MMPs)) [82, 84, 85]. Studies have shown that bacterial translocation induces pro-inflammatory responses and is associated with early death in patients with severe injury [86, 87]. Interestingly, IL-22 prevents microbial dysbiosis and promotes intestinal barrier regeneration following acute injury [81]. IL-22 has become an important target for clinical care among patients with psoriasis, ulcerative colitis, liver and pancreatic damage, and tumors [82]. These approaches show great potential for future therapeutics aimed at enhancing tissue repair.

#### Anti-inflammatory cytokines

Anti-inflammatory cytokines are immunomodulatory molecules that suppress inflammatory responses. Cytokines act synergistically with specific cytokine inhibitors and soluble cytokine receptors to modulate human immune responses. To balance and control inflammation, anti-inflammatory cytokines are produced simultaneously with pro-inflammatory cytokines. Common anti-inflammatory cytokines include IL-10, IL-11, IL-13, and TGF-β (Table 2).

**Table 2:** Cytokines with anti-inflammatory activities

Cytokines	Source	Major activities
IL-10 [61–69]	Monocytes, macrophages, and T-cells	Anti-inflammatory cytokines, inhibit Th1 cell and cytokine release, inhibit T-cell proliferation, promote B-cell differentiation, and inhibit monocyte/macrophage MHC class II expression
IL-11 [70, 71]	Stromal cells and fibroblasts	Inhibit pro-inflammatory cytokine response by monocyte/macrophages and promotes Th2 lymphocyte response and B-cell differentiation
IL-13 [79, 81]	T-cells (Th2) and mast cells	Share homology with IL-4, as well as the IL-4 receptor, attenuate monocyte/macrophage function, and promote B-cell proliferation
TGF-β [86–88]	Macrophages, T-cells, B-cells, and mast cells	Inhibit monocyte/macrophage MHC class II expression and pro-inflammatory cytokine synthesis

*IL* interleukin, *MCP* monocyte chemoattractant protein, *MIP* macrophage inflammatory protein, *TGF* transforming growth factor, *TNF* tumor necrosis factor, *MHC* major histocompatibility complex

#### IL-10

IL-10 is a typical anti-inflammatory cytokine produced by monocytes, macrophages, dendritic cells, neutrophils, T-cells, B-cells, NK cells, and so on. It can inhibit pro-inflammatory cytokines, including TNF-α, IL-1, IL-6, and IFN-γ [88], and reduce the synthesis of major histocompatibility complex II (MHCII), as well as adhesion and co-stimulatory molecules, thereby limiting effector T-cell responses [89]. Moreover, IL-10 plays an essential role in maintaining immune homeostasis during the inflammatory response. Hensler et al. evaluated the changes of IL-10 in trauma patients and found that it was significantly elevated in the plasma. They also observed that IL-10 levels elevated less dramatically in patients with only CNS injury than in patients with multiple traumas [90]. Another study compared the serum IL-10 levels of 12 patients with SIRS and 12 healthy volunteers and found that the SIRS group had higher IL-10 levels than the healthy group. Additionally, in a mouse model, intraperitoneal injection of IL-10 attenuated TNF-α-induced inflammatory stimuli in serum, suggesting that IL-10 has an antagonistic effect on TNF-α throughout the course of SIRS [91]. Moreover, researchers have found that injection of exogenous IL-10 and IL-13 could effectively improve the inflammatory response during the process of hepatic ischemia–reperfusion and significantly reduce liver cell damage. In a study on sepsis, researchers have found that the plasma level of IL-10 in patients with sepsis was associated with disease severity and intensity of the inflammatory response. The detection of high plasma levels of IL-10 in patients with sepsis may reflect a weaker inflammatory response, as well as immunosuppression [92, 93]. Another study has suggested that IL-10 enhances sepsis-related immunosuppression and predicts mortality [94]. In studies on TBI, researchers have found that IL-10 reduces the antigen presentation ability of monocytes by downregulating MHCII expression, thus reducing antigen-specific T-cell proliferation, which is considered to be the cause of immunosuppression after TBI [95, 96].

#### IL-11

Il-11 is produced by mesenchymal cells, including white blood cells, fibroblasts, epithelial cells, and osteoblasts [97]. Researchers have found that IL-11 can inhibit the inflammatory response by inhibiting the activity of NF-κB and reducing the production of inflammatory mediators, including TNF-α, IL-1β, IL-6, and IL-12 [98]. Bozza et al. found that IL-11 can be used as a direct inhibitor to Th2 cell production of cytokines and Th1 lymphocytes, limiting the differentiation of helper T-cells by modulating the cytokines produced by CD4^+^ T-cells. This increases IL-4 and IL-19 production, thereby reducing tissue damage [99]. IL-11 is usually not detected in circulation, tends to be concentrated at the site of injury, and acts primarily as an anti-inflammatory cytokine; however, it also has pro-inflammatory roles [100]. Studies have found that IL-11 can promote platelet maturation and increase platelet count [101, 102]. IL-11, IL-3, and IL-4 synergistically stimulate hematopoietic stem cell proliferation and promote erythropoiesis and differentiation. Additionally, IL-11 can regulate the expression of plasma protein genes in hepatocytes and induce acute-phase protein production [103]. In sepsis studies, IL-11 has been found to accelerate platelet production, reduce the degree of the inflammatory response, and reduce mortality in sepsis patients with thrombocytopenia [104] Moreover, Lee et al. used IL-11 to treat a mouse model of renal ischemia–reperfusion injury. Results have shown that the degree of renal tubular necrosis and infiltration of peripheral neutrophils were reduced in mice treated with IL-11, demonstrating that IL-11 has a protective effect on renal ischemia–reperfusion injury [105]. Thus, IL-11 may have protective effects on the organs of trauma patients with massive blood loss.

#### IL-13

IL-13 is a monocyte and B-cell function regulator secreted by activated T lymphocytes [106]. IL-13 has similar structural and biological effects to IL-4 and shares cell receptors [107, 108]. IL-13 also has a strong growth-promoting effect on activated B-cells. Additionally, it can downregulate the production of TNF, IL-1, IL-8, and MIP-1α through monocytes and significantly affect the expression of surface molecules in monocytes [107, 108]. In a study on trauma complications, IL-13 was considered an important regulator of ischemia–reperfusion injury and had a significant protective effect on hepatocytes and endothelial cells after liver ischemia–reperfusion. IL-13 has also been shown to significantly reduce hepatocyte injury [109]. Clausen et al. found elevated levels of IL-13 in the brain tissue and plasma in a rat model of TBI [50]. Additionally, the increase in IL-13 was related to the degree of leukopenia in patients with SIRS [110]. Another study found that IL-13 could inhibit the inflammatory injury of the lungs following the deposition of IgG immune complexes. When anti-inflammatory cytokines were administered to the lungs of rats, IL-13 exerted a strong anti-inflammatory effect [111]. Similarly, Lentsch et al. showed that exogenous IL-13 could inhibit acute lung inflammation in rats and that IL-13 antagonist treatment increased pulmonary vascular permeability, significantly increased the number of neutrophils in the bronchoalveolar lavage fluid, and caused a severe inflammatory response in rats [112]. In summary, IL-13 plays an important anti-inflammatory role in various post-traumatic inflammation processes.

#### TGF-β

TGF-β is a dimeric protein comprising three isoforms: TGF-β1, TGF-β2, and TGF-β3, each having a unique function [113]. TGF-β1, the most common isoform, is present in the cartilage, bones, skin, and intrachondral tissue and plays an important role in growth and tissue differentiation. TGF-β2 is expressed by neurons and astrocytes and plays a key role in cell proliferation. TGF-β3 is expressed in palate and lung tissues and is involved in epithelial–mesenchymal interactions [114]. In vitro, TGF-β can inhibit the release of stimulatory mediators, including IL-1, TNF-α, and HMGB1 from monocytes and macrophages, increase the production of anti-inflammatory factors, and promote the maturation of regulatory T-cells [115]. Among studies on sepsis, both animal models and clinical studies have confirmed the anti-inflammatory effects of TGF-β. TGF-β regulates the inflammatory response of sepsis by inhibiting the release of pro-inflammatory cytokines and inhibiting an excessive inflammatory response [116]. Perrella et al. reported that TGF-β also improved blood pressure and improved survival in a rat model of septic shock [117]. Moreover, compared with healthy controls, patients with sepsis had elevated TGF-β levels [118], which peaked in the early stages and were not directly related to disease severity or prognosis [119]. Furthermore, Kumar et al. found TGF-β to be cardioprotective in sepsis-induced cardiac injury [120]. However, another study demonstrated that TGF-β increased the production of pro-inflammatory cytokine TNF-α in sepsis, enhanced the synthesis of hepatic acute-phase reactive protein, and promoted the occurrence of septic shock and even death in rats [121].

#### Tissue protective and reparative functions

Type 2 immunity contributes to tissue regeneration and fibrosis following injury, with IL‑4, IL‑5, IL‑9, IL‑13, IL‑25, and IL‑33 playing a crucial role. These factors work together with immune cells, including T helper 2 cells and type 2 innate lymphoid cells, to regulate tissue repair [122, 123]. IL-25 contributes to skin healing following skin injury and could promote keratinocyte proliferation, migration, and differentiation to repair the damage [124]. Following tissue injury, damaged epithelial cells secrete IL‑25 and IL‑33. These cytokines recruit innate cells, including basophils, mast cells, and group 2 innate lymphoid cells (ILC2s), resulting in the production of the type 2 cytokines IL‑4, IL‑5, and IL‑13. These cytokines and cells engage in protective activity by reducing tissue inflammation and activating important tissue-regenerative mechanisms.

### Cytokine-based diagnosis of post-traumatic inflammatory response

Studies have shown that the pro- or anti-inflammatory cytokines can reflect the degree of tissue injury and is related to the severity, prognosis, and mortality of trauma patients [10]. For example, Volpin et al. have suggested that IL-4, IL-6, IL-8, and TGF-β could serve as potential biomarkers of a systemic inflammatory response in trauma patients [12]. Cuschieri et al. found that IL-6 levels were correlated with MODS incidence, suggesting that IL-6 could be used as a biological indicator of the prognosis of patients with severe trauma and help assess the risk of adverse outcomes in patients [125]. Some studies have also suggested that the level of cytokines is correlated with the injured organs. For example, IL-10 has been reported to be significantly higher in patients with moderate-to-severe central nervous system injury [90, 126], suggesting that changes in IL-10, combined with clinical manifestations, can be used to predict whether patients with multiple injuries also have brain injury. Furthermore, De'ath et al. noted that elevated circulating troponin levels in trauma patients accompanied by elevated TNF-α, IL-6, or IL-8 levels increased mortality risk owing to the occurrence of adverse cardiac events, suggesting that changes in cytokines in trauma patients may lead to adverse cardiac events [127].

Although cytokine measurement has demonstrated potential value in research, it has not yet been widely adopted in clinical practice. The primary reasons for this include: the majority of studies remain in the experimental phase and have not gained the broad recognition seen with tumor markers, thus necessitating more compelling research as evidential support; a lack of standardization and reference ranges, despite certain cytokines showing associations with the development of systemic inflammatory responses, their predictive value is still under investigation. More large-scale studies are needed to validate whether cytokine measurements can accurately predict disease progression and treatment response in patients. In addition, the adoption of any new diagnostic or monitoring tool in clinical practice must consider cost-effectiveness. It is crucial to assess whether the information gained from cytokine measurements justifies the associated costs. Future research should include health economics analyses to determine whether cytokine-guided strategies are cost-effective in severe trauma management, taking into account potential reductions in hospital stay, resource utilization, and improved patient outcomes.

### Cytokine-based therapy for post-traumatic inflammatory response

Cytokine therapy has made some advances in trauma services and injury management. Blocking the signaling pathways of specific cytokines in the early stage of trauma may attenuate excessive inflammatory responses. Researchers have looked for solutions involving pro-inflammatory cascade initiators, regulatory cytokines, and the elimination of pro-inflammatory cytokines. Others have studied the effects of genetic polymorphisms on the development of SIRS [128]. Treatment by blocking cytokines has long been a popular area of research, with many studies exploring autoimmune diseases and tumors. Currently, monoclonal antibody drugs targeting cytokines have been approved by the Food and Drug Administration (FDA) in the United States to treat patients with advanced melanoma and renal cell carcinoma [129]. It is expected that these strategies will be validated for the treatment of trauma patients.

Strategies for regulating cytokine function in patients with severe trauma include antagonism or filtration [39]. Research on drug regulation mainly includes antibody inhibition and cytokine pretreatment. In a study on traumatic hemorrhagic shock, Frink found that the expression of keratinocyte-derived chemokine (KC) was higher in traumatic hemorrhage models than in cohort comprising healthy subjects. The use of anti-KC antibodies reduced pulmonary and liver edema in the models by reducing bleeding and neutrophil infiltration after resuscitation, thereby relieving organ damage and improving the survival rate. These findings suggest that critical factors can be identified to improve treatment efficiency for hemorrhagic shock [130]. Moreover, in patients with sepsis, serum TNF-α and IL-6 levels were significantly lower, organ failure scores improved more rapidly, and mortality was slightly lower in the afelimomab (monoclonal anti-TNF-α) group than in the placebo group [131]. However, in clinical trials, IL-1 receptor antagonists (anakinra) for patients with sepsis have not been effective in reducing sepsis-related mortality. In contrast, anakinra specifically improved the concurrent hepatobiliary dysfunction and survival in patients with sepsis and disseminated intravascular coagulation [132]. Furthermore, Li et al. found that IL-33 was specifically found in mice with liver ischemia–reperfusion injury and that IL-33 pretreatment could reduce the severity of liver injury in mice [12]. Similarly, Wei et al. found that, following the injection of IL-5 in rats with lung injury, the degree of injury and water content of the lung tissue was significantly lower in the lung injury group than in the control group. They concluded that IL-5 can reduce lung injury by regulating the immune response and inhibiting a systemic inflammatory response [133].

As for cytokine removal strategies, the use of continuous hemofiltration and hemodialysis to effectively remove cytokines is the current standard method [39]. Optional strategies for cytokine adsorption include synthetic polymer resin, immobilized antibody system, activated carbon, and polymyxin B immobilized fiber column, which all boast good experimental results. For example, a previous study demonstrated that activated carbon could absorb almost 100% of lipopolysaccharide (LPS), IL-Ra, IL-1β, IL-8, and IL-1a and 40% of TNF-α in plasma. Smaller IL-6 and IL-1β molecules could be removed entirely within 5 min [134, 135].

While reducing the inflammatory response may seem beneficial, it is crucial to consider the overall impact on the body and whether it leads to positive outcomes. While these percentages suggest that this method can reduce the inflammatory response, whether it is beneficial to the body remains uninvestigated. Methods to remove cytokines associated with inflammation are gradually being explored, though clinical evidence is currently lacking.

The various methods mentioned above have demonstrated potential effects in laboratory and small-scale clinical studies. However, they lack confirmation of their safety and efficacy through large-scale, randomized controlled clinical trials. Additionally, the use of drugs to antagonize or filter out inflammatory cytokines may entail certain risks, such as an increased risk of immunosuppression and infection. These potential risks necessitate careful consideration, particularly in cases of severely traumatized patients with compromised immune systems.

## Discussion

SIRS resulting from severe trauma and the subsequent development of MODS are primary factors contributing to poor patient prognosis and treatment efficacy. Unfortunately, there is currently no widely recognized aggressive treatment approach that consistently benefits these patients [5].

Currently, cytokine therapies are generally more applicable to localized issues, such as post-traumatic arthritis. These treatments are typically directed at specific tissues or organs rather than being systemic in nature. For example, patients with traumatic arthritis may receive intra-articular cytokine therapy, including the injection of one or more cytokines such as tumor necrosis factor inhibitors, directly into the affected joint to reduce inflammation, promote repair, and improve joint function [136]. While cytokine inhibition therapy can produce significant effects in some cases, its efficacy in the treatment of systemic inflammatory responses resulting from trauma is still not sufficiently clear and has not yet garnered adequate evidence-based support within the field of evidence-based medicine, making conclusions in such instances occasionally equivocal. The use of systemic cytokine inhibitors after severe trauma should be approached with extreme caution. Consideration must be given to the overall regulation of the immune system to avoid compromising its function and increasing the risk of infection. When using cytokine inhibitors, close monitoring of the patient's immune status and infection risk is essential, along with regular medical assessments and checks.

The important factor causing SIRS and MODS after trauma is excessive release of cytokines. With the exploration of the disease, scientists have updated the concept of sirs out of control to the concept of “cytokine storm”, and summarized the clinical problems related to sirs and mods. Cytokine storm was reviewed in The New England Journal of Medicine and defined as a life-threatening systemic inflammatory syndrome characterized by immune dysregulation characterized by persistent systemic symptoms, such as fever and systemic inflammatory response, as well as multiple organ dysfunction. Cases of cytokine storm can progress rapidly to disseminated intravascular coagulation with vascular occlusion or massive bleeding, as well as dyspnea, hypoxemia, and patients can rapidly go into shock and death [25]. Based on the different mechanisms of various diseases, scientists in different fields have proposed cytokine storm in different diseases. Such as the unique cytokine storm in COVID-19 patients and the cytokine storm in cancer patients. Many high-quality studies have been published in the field of COVID-19 and oncology. However, at present, there are few studies describing the integration of cytokines after trauma. This stands as one of the future directions for the research.

Omics research, including genomics, transcriptomics, proteomics, and single-cell omics create a more comprehensive view of the systemic response to injury [137]. Bonaroti et al. have described the circulating immune mediator landscape in the early stage after trauma. Using plasma proteomics, the authors reported an early release of cytokines and delayed increases in cytokine levels, which are associated with trauma outcomes, in patients that remain critically ill [138]. St John et al. presented the changes in the plasma proteomic profile of platelet dysfunction after trauma. They found the proteins with the most profound changes, which provided insight into the pathophysiology of platelet dysfunction after trauma [139]. Krocker et al. identified protein s and pathways that are likely to be driving the pathologic processes underlying the endotheliopathy of trauma, with the corresponding proteomic profile providing targets for pharmacological therapeutics [140]. A multi-omics analysis of large patient populations provides new possibilities for the diagnosis and treatment of trauma patients as it identifies specific markers, which helps provide more targeted interventions for trauma patients. Our understanding of post-traumatic inflammation will be strengthened with the development of omics techniques. Omics studies have provided a lot of information, especially some that could not have been attained before. Nonetheless, these results still need to be verified by exact experiments and clinical research before they can be truly translated to clinical treatment.

This review only provides a short overview of selected cytokines that are important for injury response; however, cytokine cascades and interaction networks are complex. To better describe complex signaling following traumatic injury, researchers have tried multiplexing techniques to measure these cytokines simultaneously. Multiplexing strategies with computational methods, including principal component analysis (PCA), dynamic network analysis (DyNA), and dynamic Bayesian network (DyBN) inference [141, 142], have been widely used to help better describe complex signaling as a whole. PCA was used to define the principal characteristics of a multifaceted inflammatory response over time, including the use of individual-specific PCA with hierarchical clustering. Schimunek et al. segregated trauma patients into three core endotypes based on early inflammatory mediator networks, which are associated with the inflammatory response and multiple organ dysfunction (MOD), and the endotypes exhibiting significant differences in requirement for mechanical ventilation, duration of ventilation, and MOD over 7 days [142]. DyNA aims to define the cytokines’ time-evolving networks of systemic inflammation [141]. Abboud et al. analyzed cytokines and other inflammatory mediators assessed within the first 24 h and over 7 days using computational modeling to infer the dynamic networks of inflammation. They found two disparate dynamic networks of systemic inflammation, which predict unequivocal endpoints of post-traumatic outcomes (life or death) [143]. DyBN was used to explore mechanisms inferred from dynamic trajectories of inflammatory cytokines and delineate potential feedback structures [144]. DyBN combined with DyNA tracked the evolution of inflammatory networks, identified the relationship between individual cytokines over time, and has yielded information on network complexity [145, 146]. Using DyBN inference, Almahmoud et al. showed that IL-6 production in blunt trauma survivors was affected by monocyte chemotactic protein CCL2, IFN-γ, and IP-10 [147]. Zaaqoq et al. found that the systemic inflammatory responses of patients with blunt trauma and that of patients with traumatic spinal cord injury are significantly different. Using a novel dynamic network inference algorithm, they suggested that the chemokine IP-10 is a potential driver of IL-10 and morbidity in trauma patients [146]. Multiplexing strategies display the potential utility of identify key inflammatory drivers. These methods help us gain a global perspective on the interaction of cytokines and systemic inflammation and provide us a new idea for treating patients with severe trauma.

While many valuable findings have been uncovered in this field of research, several shortcomings remain. First, different types of cells produce various cytokines that also have varying selectivity to different receptors, forming a complex functional network. The effects of cytokines are often pleiotropic and synergistic and differ according to cell type. Thus, a single molecule or pathway cannot be integrated globally, and cytokine function cannot be evaluated solely by promoting or inhibiting inflammation. Second, the concentration of pro- and anti-inflammatory cytokines is usually measured by the concentration of the corresponding factors in the peripheral blood. However, in practice, the concentration and function of cytokines in local tissue or the microenvironment differ from those in the peripheral blood. Finally, whether the proportion of cytokines removed is controllable, and what proportion should be removed to retain the beneficial effects of inflammation while avoiding the damage caused by excessive inflammation is yet to be determined. This review only provides a short overview of select cytokines that are important for inflammatory reactions in the body.

## Conclusions

The immune system response of patients with severe trauma is complex. Patients with severe trauma exhibit variable and either overly exuberant or overly damped responses that likely drive adverse clinical outcomes. Cytokines play an important role in the post-traumatic inflammatory response and immune monitoring. More comprehensive and in-depth clinical and experimental studies are needed to reveal the cytokine function and interaction network fully.

## Data Availability

All data were obtained from public datasets.
